# Reactive Human Plasma Glutathione Peroxidase Mutant with Diselenide Bond Succeeds in Tetramer Formation

**DOI:** 10.3390/antiox11061083

**Published:** 2022-05-29

**Authors:** Zhenlin Fan, Qi Yan, Jian Song, Jingyan Wei

**Affiliations:** 1 College of Pharmaceutical Science , Jilin University, Changchun 130021, China; fanzl11@mails.jlu.edu.cn (Z.F.); qiyan20@mails.jlu.edu.cn (Q.Y.); 2 College of Electronic Science and Engineering, Jilin University, Changchun 130000, China; 3 Key Laboratory for Molecular Enzymology and Engineering of the Ministry of Education, Jilin University, Changchun 130000, China

**Keywords:** glutathione peroxidase, selenoprotein, diselenide, enzyme kinetics, protein conformation

## Abstract

Plasma glutathione peroxidase (GPx3) belongs to the GPx superfamily, and it is the only known secreted selenocysteine (Sec)−containing GPx in humans. It exists as a glycosylated homotetramer and catalyzes the reduction of hydrogen peroxide and lipid peroxides, depending on the Sec in its active center. In this study, a previously reported chimeric tRNA^UTuT6^ was used for the incorporation of Sec at the UAG amber codon, and the mature form of human GPx3 (hGPx3) without the signal peptide was expressed in amber−less *E. coli* C321.ΔA.exp. Reactive Sec−hGPx3, able to reduce H_2_O_2_ and tert−butyl hydroperoxide (*t*−BuOOH), was produced with high purity and yield. Study of the quaternary structure suggested that the recombinant Sec−hGPx3 contained an intra−molecular disulfide bridge but failed to form tetramer. Mutational and structural analysis of the mutants with three Cys residues, individually or jointly replaced with Ser, indicated that the formation of intra−molecular disulfide bridges involved structure conformational changes. The secondary structure containing Cys77 and Cys132 was flexible and could form a disulfide bond, or form a sulfhydryl–selenyl bond with Sec49 in relative mutants. Mutation of Cys8 and Cys132 to Sec8 and Sec132 could fix the oligomerization loop through the formation of diselenide bond, which, in turn, facilitated tetramer formation and noticeably improved the GPx activity. This research provides an important foundation for the further catalysis and functional study of hGPx3.

## 1. Introduction

Glutathione peroxidase (GPx) is a well−known superfamily that protects cells from oxidative stress through the reduction of hydroperoxides to their corresponding alcohols. In humans, eight members of the GPx family were identified, five of which are selenoproteins, whose catalysis depends on selenocysteine (Sec) in their active center [1,2]. Human plasma GPx (hGPx3) was first extracted from plasma and identified as a glycosylated homotetramer [3,4,5]. It is primarily expressed in the proximal convoluted tubules of the kidneys and secreted into the blood stream [6,7]. It was found that GPx3 could bind to the basement membranes of many tissues [8,9] and is catalyzed at the binding site, as the physiological low concentration of GSH cannot provide enough electron donors for the efficient reduction of hydroperoxides by GPx3. Thioredoxin (Trx) and glutaredoxin (Grx) were shown to be efficient electron donors for GPx3 [10], unlike the other Sec−containing GPx family members. The function of GPx3 was still elusive, and it may act not only as a hydroperoxide reducing agent, but also as a hydroperoxide sensor.

The cDNA sequence of GPx3 shows that the natural hGPx3 contains a signal peptide at the N−terminus. The prediction of the N−terminal residue is the 25th residue, while it is the 29th residue in the crystal structure of the natural GPx3 [11]. The crystal structure of hGPx3 shows that it has an external N−terminus and C−terminus, unlike bovine GPx1. The Cys8 in the external N−terminus is thought to form an intra−molecular disulfide bond with Cys132 in the C−terminus of the third α−helix [11], which contributes to the stability of the long oligomerization loop for the formation of tetramer. This disulfide bond was detected through the non−reducing SDS−PAGE analysis of natural hGPx3 [12] and recombined hGPx3 expressed in mammalian cells [13]. The formation of disulfide bonds rarely takes place in Sec−GPx, but is a necessary process for the catalysis of Cys−GPx using Trx as an electron donor [14,15].

The heterologous expression of mammalian selenoproteins in *E. coli* is difficult because of the differences in the selenocysteine decoding mechanisms between the bacteria and eukaryotes [16,17,18]. In our previous study, we succeeded in promoting the expression of an hGPx3 mutant with all of its Cys replaced with Ser, using the Cys auxotrophic *E. coli* strain [19]. The hGPx3 mutant is a monomeric protein, and is reactive with GSH as a reduction substrate; however, the role of Cys contributing to its structure and GPx activity cannot be studied using this method. A recently developed method using EF−Tu compatible tRNA (tRNA^UTu^) to encode UAG as Sec facilitates the expression of selenoproteins [20]. The tRNA^UTu^ was constructed based on tRNA^Ser^, by replacing the acceptor stem of tRNA^Ser^ with that of tRNA^Sec^, and the anticodon was changed to CUA to encode UAG as Sec. Based on tRNA^UTu^, the tRNA^UTuT6^ was developed and demonstrated the efficiency of the incorporation of Sec at the UAG codon in amber−less *E. coli* C321.ΔA.exp [21], as high as that mediated by tRNA^UTuX^ [22] in the same expression system.

In this study, tRNA^UTuT6^ was used for the expression of Sec−containing hGPx3 (Sec−hGPx3) in amber−less *E. coli* C321.ΔA.exp. The recombinant Sec−hGPx3 containing intra−molecular disulfide bonds failed to form tetramer. Various Cys residues and Sec49 in hGPx3 were mutated to Ser to identify which residues participate in the formation of disulfide bonds, using non−reducing SDS−PAGE analysis. The results showed that the formation of disulfide bonds and selenyl–sulfhydryl bond was complicated due to the flexible secondary structure containing Cys. Finally, we found that when Cys8 and Cys132 were mutated to Sec8 and Sec132, the mutant Sec−hGPx3−C8/132U succeeded in tetramer formation, and the GPx activity was noticeably improved. The steady kinetic study of Sec−hGPx3−C8/132U showed that it obeys saturation ping pong kinetics with respect to GSH, the same as when purified from human plasma [5]. This study should facilitate further progress in understanding the catalysis and function of hGPx3.

## 2. Experimental Procedures

### 2.1. Plasmids and E. coli Strains

The *E. coli* strains and plasmids developed and used in this study are listed in Table 1. The amber−less *E. coli* strain C321.ΔA.exp [23] was obtained from Addgene (49018) and was used for the selenoprotein expression. Plasmid pN565 [24] was a gift from Christopher Voigt (Addgene plasmid # 49990) and was transformed into C321.ΔA.exp for T7 RNA polymerase expression under Isopropyl β−D−1−thiogalactopyranoside (IPTG) induction. The plasmids pACYC−[*E. coli selA+M. jannaschii pstk*] (pACYC−*selA/pstk*) and pGFIB−tRNA^UTu^ were generous gifts from Dieter Söll [20].

### 2.2. Cloning Procedures

The *hGPx3* gene without the N−terminal signal sequence (nucleic acids 1–72) was synthesized and subcloned into the BamHI and HindIII sites of pUC57 by Sangon Biotech, yielding pUC57−*hGPx3*. The expression plasmid pRSF−*hGPx3* was constructed by subcloning the BamHI/HindIII fragment from pUC57−*hGPx3* into the BamHI and HindIII sites of pRSF−Dute1. The Sec codon in pRSF−*hGPx3* was point mutated to TAG, using the primer pair TAG−F and TAG−R to generate pRSF−*hGPx3_49TAG_*. The plasmid pRSF−*hGPx3_49TAG_* was used as the template for the site−directed mutagenesis reactions, and the mutagenic primers used in this study are listed in Table S1 (Supplementary Materials). The Sec to Ser mutation was carried out, using the same method with the primers TCG−F and TCG−R. The genes for the short isoform of hGPx3 and its mutants, without the external N−terminal peptide QEKSKMDCHGGIS, were amplified from the plasmids pRSF−*GPx3_49TAG_*, pRSF−*GPx3_49TAG_−C77S*, pRSF−*GPx3_49TAG_−C132S*, and pRSF−*GPx3_49TAG_*−*C8/77/132S,* with the primers shGPx3−F and hGPx3−R. After digestion with BamHI and HindIII, the PCR products were ligated into the BamHI and HindIII sites of pRSFDuet−1, yielding pRSF−*shGPx3_49TAG_*, pRSF−*shGPx3_49TAG_−C77S*, pRSF−*shGPx3_49TAG_−C132S*, and pRSF−*shGPx3_49TAG_−C77/132S*. All of the constructs were verified by DNA sequencing.

### 2.3. Protein Expression and Purification

Protein expression and purification were carried out, using a previously described method [21]. The purified proteins in this study were described in Table 2. Plasmids carrying genes encoding hGPx3 and its mutants were transformed into the *E. coli* strain C321.ΔA.exp, which was co−transformed with the plasmids pN565, pGFIB−tRNA^UTuT6^, and pACYC−*selA/pstk*. Starting from a single colony after transformation, cells were grown at 37 °C in 1 mL of LB selective medium supplemented with ampicillin (100 μg/mL), kanamycin (25 μg/mL), chloramphenicol (34.4 μg/mL), and spectinomycin (90 μg/mL) on a shaker. Then, 200 μL of overnight culture was added to 200 mL of LB medium supplemented with ampicillin (100 μg/mL), kanamycin (25 μg/mL), chloramphenicol (34.4 μg/mL), and spectinomycin (90 μg/mL), with or without sodium selenite (50 µM), followed by incubation at 37 °C for several hours. When the optical density of the culture at 600 nm reached 1.2–1.5, the temperature was decreased to 20 °C, and 4 h later, protein expression was induced with 100 µM IPTG, followed by continued incubation for 18 h at 20 °C. Cells were collected by centrifugation (5000× *g* for 5 min at 20 °C), and the cell pellet was resuspended in 10 mL of lysis buffer (50 mM sodium phosphate, 300 mM NaCl (pH 7.4), and 30 mM imidazole). After lysis by sonication, the lysate was clarified by centrifugation (20,000× *g*, 10 min), passed through a 0.2 μm filter, and purified using an immobilized metal−affinity chromatography purification system with Ni–NTA resin. The protein concentration was determined by the Bradford method, using bovine serum albumin as a standard.

### 2.4. SDS−PAGE Analysis

For purity analyses, 3 μg purified GPx samples were separated on 12% SDS−PAGE gels after boiling in SDS loading buffer (10 mM Tris−HCl, 4% SDS, 5% β−mercaptoethanol (β−ME), 15% glycerol, and 0.002% bromophenol blue at pH 6.8) for 5 min. The protein bands were visualized by staining with Coomassie Brilliant Blue R−250. Non−denatured samples were loaded without boiling treatment. To prepare non−reduced samples, β−ME was omitted from the SDS loading buffer and the samples were loaded without boiling treatment.

### 2.5. GPx Activity Assays

The GPx activities of recombinant Sec−hGPx3 and its mutants were determined, using a previously described method [25]. Sodium phosphate (50 mM pH 7.4), EDTA (1 mM), GSH (1 mM), NADPH (0.25 mM), glutathione reductase (GR) (1 U), and the samples were mixed in a cuvette at 37 °C. The reagent mixture was incubated for 3 min at 37 °C, and the reaction was initiated by the addition of 60 μM H_2_O_2_ or tert−butyl hydroperoxide (*t*−BuOOH) (final concentration), in a total volume of 0.5 mL. GPx activity was determined by measuring the decrease in NADPH absorption at 340 nm over time. Activity units (U) were defined as the amount of enzyme necessary to oxidize 1 μmol NADPH per min at 37 °C. The specific activity was expressed in activity units per milligram (U/mg) of protein. Samples were run in triplicate, and the values were averaged.

### 2.6. Effect of Temperature and pH on GPx Activity

The optimal temperature and the pH for GPx activity of Sec−hGPx3−C8/132U were determined by performing enzymatic assays at different temperatures (20 to 56 °C) and pH levels (6 to 12), respectively. The pH was detected after the reaction stopped.

### 2.7. Steady−State Kinetics of hGPx3

Steady−state kinetics were carried out, following the method described above [26]. The steady−state parameters for Sec−hGPx3−C8/132U were determined, using the same assay with a variable concentration of *t*−BuOOH, while GSH was kept at 1 mM, 1.5 mM, 3.0 mM, 5.0 mM, and 10.0 mM, respectively. GPx activities were measured, using the same method as described above, at 37 °C and pH 7.4. Kinetic data values were calculated from the Dalziel coefficients, as described previously [14].

### 2.8. Molecular Modelling

The qualitative molecular modeling was performed with the Insight II package, version 2000 (Accelrys, San Diego, CA, USA). The X−ray crystal structure of hGPx3 with Sec−49 mutated to Gly (Protein Data Bank ID: 2R37) was used for the starting coordinates. The 3D structure of the Sec−hGPx3 with the external N−terminal peptide was refined by MD simulations and analyzed with Profile−3D and PROCHECK, following the method as described previously [27,28]. The obtained models were energy minimized, using the method of conjugate gradient (300 steps) minimization, and then a molecular dynamics (MD) simulation was performed for 1 ns simulations at a constant temperature 298 K to examine the quality of the model structures. TIP3P water was used to construct a 20 Å water cap from the center of mass of each model, and then the models were subsequently energy minimized until the root mean−square gradient energy was lower than 0.001 kcal/mol Å. All of the calculations were performed using the Discover 3 software package (Insight II, Version 2000, Accelrys, San Diego, CA, USA)[29,30]. The molecular visualization system, PyMOL, was used to obtain the 3D−predicted structures.

## 3. Results

### 3.1. Expression and Purification of Sec−hGPx3 with Intra−Molecular Disulfide Bond Formation

**Figure 1:**
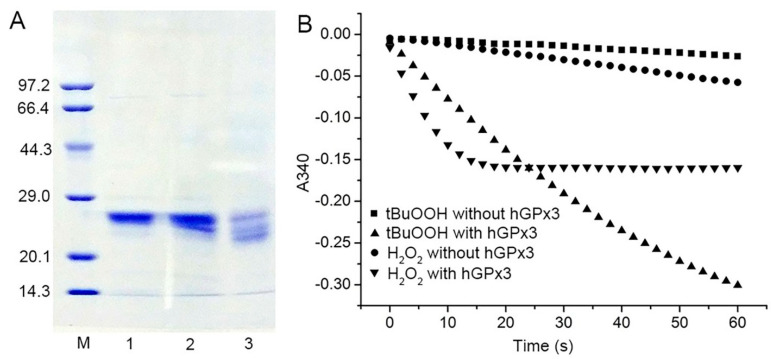
Expression, purification, and enzymatic activity of Sec−hGPx3; (**A**) SDS−PAGE analysis of purified Sec−hGPx3 under reducing conditions (Lane 1), reducing conditions without heat−denaturation (Lane 2) and non−reducing conditions without boiling treatment (Lane 3). M, molecular mass markers; (**B**) Enzymatic activity of Sec−hGPx3 (2.5 μg) was assessed through the decrease of absorbance at 340 nm over time upon the addition of tert−butyl hydroperoxide *(t*−BuOOH) (60 μM, triangle) or H_2_O_2_ (60 μM, upside−down triangle) to a cuvette containing glutathione reductase (GR) (1 U), GSH (1 mM), and NADPH (0.25 mM). Control reactions were spontaneous reduction of H_2_O_2_ (circle) and *t*-BuOOH (squares) without Sec−hGPx3.

The amber−less *E. coli* strain C321.ΔA.exp, with all of its endogenous 321 UAG amber codons replaced with UAA and its UAG−specific release factor 1 deleted, was used for the Sec encoding by the UAG codon. Recombinant Sec−hGPx3 was expressed, using tRNA^UTuT6^ to suppress the UAG amber codon for Sec in amber−less *E. coli* C321.ΔA.exp. The yield of Sec−hGPx3 was approximately 2.5 mg/L of culture. As shown in Figure 1A, under reducing conditions with or without heat−denaturation, Sec−hGPx3 migrated as a single band with apparent molecular masses of about 25 kDa on SDS−PAGE, while it migrated as a triple band, with two molecular mass bands migrating faster under non−reducing conditions, suggesting that intra−molecular disulfide bonds were formed in Sec−hGPx3.

The specific activity of Sec−hGPx3 was determined, using a GR−coupled assay with H_2_O_2_ or *t*−BuOOH as substrates (Figure 1B), which revealed significant GPx activity. Under the assay conditions with 60 μM H_2_O_2_ and 1 mM GSH, the specific activity was 20.9 U/mg protein, showing approximately the same specific activity as native GPx3 reported in 1987 [3,4,5], but definitely lower activity compared with that reported by Takebe et al. [26].

As shown in Figure 1B, the slope for the reduction of H_2_O_2_ by Sec−hGPx3 became flat because H_2_O_2_ was eliminated by Sec−hGPx3 and the reaction stopped. A reduction of 30 nmol (60 μM in 0.5 mL solution) hydroperoxide could produce about 30 nmol GSSG and consume 30 nmol NADPH, the absorption value at 340 nm decreased by about 0.37. The faster initial catalytic speed for H_2_O_2_ compared to *t*−BuOOH made the final absorption value at 340 nm approximately −0.16 for the reduction of H_2_O_2_.

### 3.2. Analysis of Disulfide Bond Formation in Sec−hGPx3

**Figure 2:**
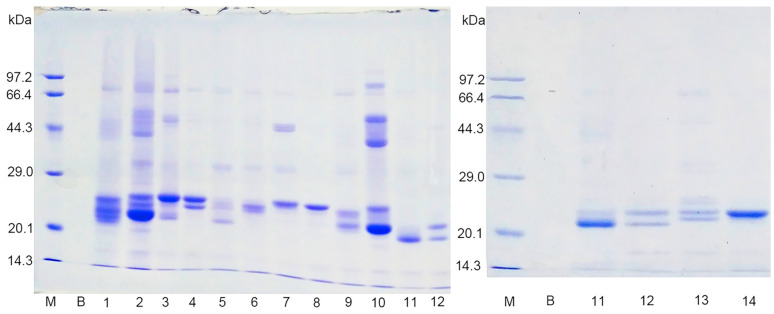
SDS−PAGE analysis of hGPx3 and hGPx3 mutants under non−reducing conditions without boiling treatment. Lanes are as follows: M: marker; B: blank; 1: Sec−hGPx3; 2: Sec−hGPx3−C8S; 3: Sec−hGPx3−C8/77S; 4: Sec−hGPx3−C8/132S; 5: Sec−hGPx3−C77S; 6: Sec−hGPx3−C132S; 7: Sec−hGPx3−C77/132S; 8: Sec−hGPx3C8/77/132S; 9: Ser−hGPx3; 10: Ser−hGPx3−C8S; 11: Sec−shGPx3; 12: Sec−shGPx3−C77S; 13: Sec−shGPx3−C132S; 14: Sec−shGPx3−C77/132S.

We further investigated which Cys residues participated in the formation of intra−molecular disulfide bonds in Sec−hGPx3. The three Cys residues were mutated individually or jointly to Ser for analysis of the disulfide bond formation in the mutants. The non−reducing SDS−PAGE results for the Sec−hGPx3 mutants showed that the intra−molecular disulfide bond formation was very complicated. When Cys8 was mutated to Ser, most of the protein migrated as a single lower band (Figure 2, Lane 2). This result was also observed for the short isoform of Sec−hGPx3 (Sec−shGPx3) without Cys8 (Figure 2, Lane 11). These results suggested that the disulfide bond was formed between Cys77 and Cys132, and that its formation was facilitated by the absence of Cys8. It was surprising that the disulfide bond still existed with only one Cys remaining, which suggested that Sec49 might form a selenyl–sulfhydryl bond with Cys77 or Cys132. The upper and lower bands were observed for Sec−hGPx3−C8/77S, Sec−hGPx3−C77S, and Sec−shGPx3−C77S (Figure 2, Lanes 3, 5, and 12, respectively), while the middle band was not observed. The condition was different for Sec−hGPx3−C8/132S, Sec−hGPx3−C132S, and Sec−shGPx3−C132S (Figure 2, Lanes 4, 6, and 13, respectively); the upper and middle bands were observed and the lower band was not observed. These results suggested that Cys77 might participate in the formation of a bond corresponding to the middle band, while Cys132 might participate in the formation of a bond corresponding to the lower band.

**Figure 3:**
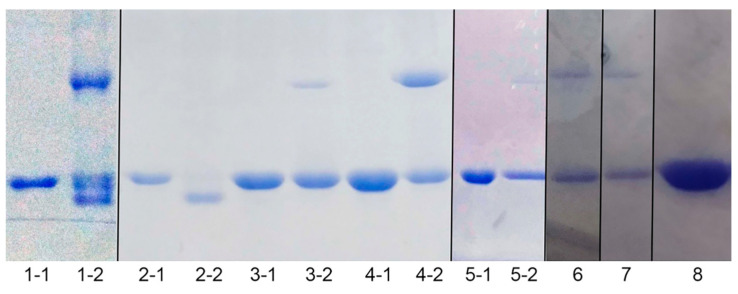
SDS−PAGE analysis of hGPx3 (U49S) and hGPx3 (U49S) mutants under reducing conditions with boiling treatment or non−reducing conditions without boiling treatment. Lanes are as follows: 1−1, reduced hGPx3 (U49S); 1−2, non−reduced hGPx3 (U49S); 2−1, reduced hGPx3 (U49S)−C8S; 2−2, non−reduced hGPx3 (U49S)−C8S; 3−1, reduced hGPx3 (U49S)−C8/77S; 3−2, non−reduced hGPx3 (U49S)−C8/77S; 4−1, reduced hGPx3 (U49S)−C8/132S; 4−2, non−reduced hGPx3 (U49S)−C8/132S; 5−1, reduced hGPx3 (U49S)−C77S; 5−2, non−reduced hGPx3 (U49S)−C77S; 6, non−reduced hGPx3 (U49S)−C132S; 7, non−reduced hGPx3 (U49S)−C77/132S; 8, non−reduced hGPx3 (U49S)−C8/77/132S.

Ser−hGPx3 and Ser−hGPx3−C8S were expressed without sodium selenite supplementation, in order to detect whether Sec participated in the formation of the selenyl–sulfhydryl bond. The middle band was not observed for Ser−hGPx3−C8S (Figure 2, Lane 10), indicating that Sec49 might form a selenyl–sulfhydryl bond with Cys77. Sec49 was mutated into Ser for non−reducing SDS−PAGE analysis (Figure 3). The hGPx3 (U49S) and hGPx3 (U49S)−C8S were found to form intra−molecular disulfide bonds. Except for hGPx3 (U49S)−C8/77/132S, the other mutants formed inter−molecular disulfide bonds instead of intra−molecular disulfide bonds, indicating that Sec49 was important for the structure. The same results were also observed for shGPx3 when Sec49 was mutated to Ser (Figure S1, Supplementary Materials). Taken together, we can conclude that Cys77 and Cys132 could form an intra−molecular disulfide bond in Sec−hGPx3, the formation of which was facilitated in the absence of Cys8, and Sec could form selenyl–sulfhydryl bonds with both Cys77 and Cys132 when one of them was mutated to Ser.

**Figure 4:**
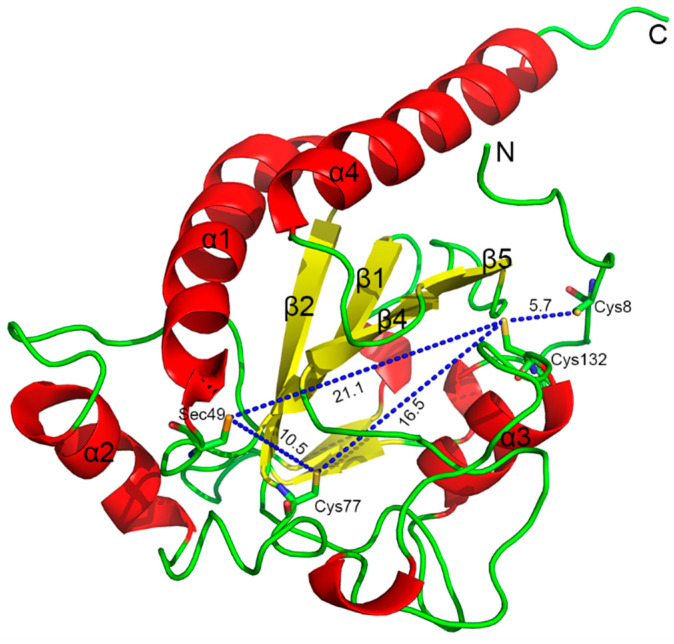
The overall structure of Sec−hGPx3 with the external N−terminal peptide; Cys8, Cys77, Cys132, and the catalytic center Sec49 are shown as sticks and labeled. The distances were measured and are shown by blue dotted lines and labeled. Cartoon representations were generated in PyMOL.

The structure of Sec−hGPx3 with an external N−terminal peptide was modeled from the crystal structure of the Sec to Gly mutant of hGPx3 (Protein Data Bank ID: 2R37), with reference to the crystal structure of GPx3 purified from human plasma [11]. The result of molecular modeling of Sec−hGPx3 is shown in Figure 4. The distance from Cys132 to Cys77 and Sec49 was calculated to be 16.5 Å and 21.1 Å, respectively; thus, disulfide or selenyl–sulfhydryl bonds could not be formed without a large conformational change in the secondary structural architecture. Cys77 is conserved in both SecGPx and CysGPx and occupies a prominent position at the C−terminal end of β2. Cys132 is conserved in CysGPx, but is not present in SecGPx members such as GPx1 and GPx2; it occupies the position at the C−terminal end of α3. The distance between Cys77 and Sec49 was 10.5 Å, and the two could form a selenyl–sulfhydryl bond after conformational changes. When Cys8 was mutated or deleted, the loop between α3 and β4 might become much more flexible in dynamic situations, making the distance between Cys132 and Cys77 become much shorter, enabling the formation of a disulfide bond. Such disulfide bond formation is often found in CysGPx and it was proven to be necessary for the catalytic process [14,15]. The evolutionary relationship between hGPx3 and CysGPx requires further investigation.

### 3.3. Effect of Cys Mutation on the Catalytic Activity of hGPx3

**Figure 5:**
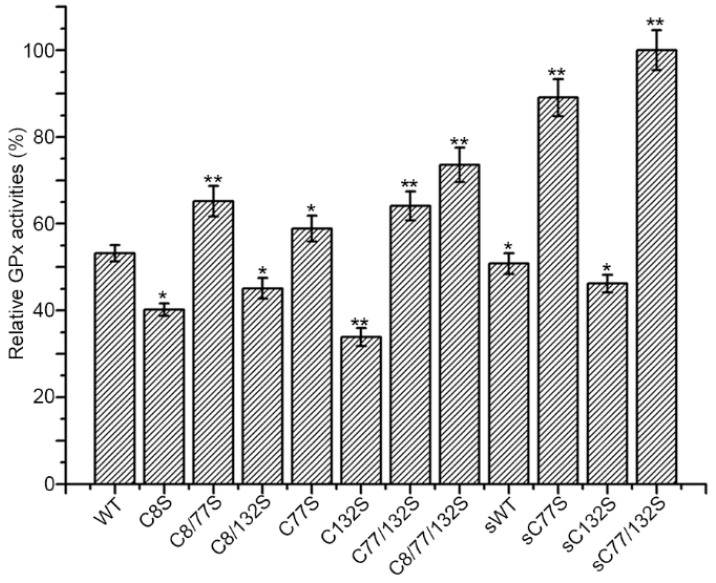
Effect of point mutations on the GPx activity: The GPx activities of WT (Sec−hGPx3) and mutants were analyzed under the same conditions as described in the legend of Figure 1, except 500 μM H_2_O_2_ was used. The activity of Sec−shGPx3−C77/132S (56.2 U/mg) was defined as 100%. Student’s *t*−test was used for statistical analysis. The data are presented as the mean ± SD (*n* = 3); * *p* < 0.05, ** *p* < 0.01.

We further detected the GPx activity of each mutant toward H_2_O_2_ (500 μM), in order to evaluate the effects of the Cys mutation. All of the Sec−hGPx3 mutants were able to reduce H_2_O_2_, using GSH as a redox donor (Figure 5). The mutant Sec−hGPx3−C8/77/132S showed the highest GPx activity compared to the other variants with the external N−terminal peptide. The GPx activity was further improved for Sec−shGPx3−C77/132S when the external N−terminal peptide was deleted. The complicated formation of intra−molecular disulfide bonds deceased the GPx activity.

In Sec−hGPx3−C8/77S, Sec−hGPx3−C77S, Sec−hGPx3−C77/132S, Sec−hGPx3−C8/77/132S, Sec−shGPx3−C77S, and Sec−shGPx3−C77/132S, Cys77 was replaced with Ser, and the GPx activities were higher when compared to the mutants that retained Cys77. The formation of a selenyl–sulfhydryl bond between Sec49 and Cys77 may have directly lowered the activity.

### 3.4. Mutation of Cys8 and Cys132 to Sec8 and Sec132 Promotes Tetramer Formation

**Figure 6:**
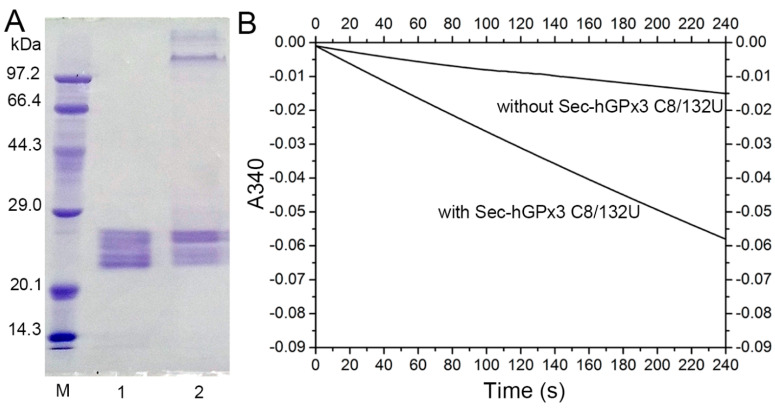
Expression of enzymatically active Sec−hGPx3−C8/132U. (**A**) SDS−PAGE gel analysis of Sec−hGPx3−C8/132U; Lane 1: reduced Sec−hGPx3−C8/132U; Lane 2: non−reduced Sec−hGPx3−C8/132U; (**B**) Reduction of *t*−BuOOH catalyzed by Sec−hGPx3−C8/132U with 10 μM GSH. The reactions were performed with 1 mM EDTA, 0.25 mM NADPH, 0.3 mM *t*−BuOOH, 1 U GR, and 10 μM GSH, 0.4 μM GPx3 (final concentration) was added to the experimental group.

The failure to form a disulfide bond between Cys8 and Cys132 might be the main reason for the complicated non−specific disulfide bond formation and the failure of tetramer formation. Given that diselenide bonds are more stable than disulfide bonds and have a lower redox potential [31], a diselenide bond was introduced into Sec−hGPx3 by the mutation of Cys8 and Cys132 to Sec8 and Sec132. The SDS−PAGE analysis showed that Sec−hGPx3−C8/132U without heat−denaturation treatment partially formed a tetramer (Figure 6A). The GPx activity was also improved after diselenide bond formation. Under the assay conditions with 60 μM H_2_O_2_ and 1 mM GSH, the specific activity of Sec−hGPx3−C8/132U was 31.2 U/mg protein. Our previous studies on the expression of Sec−hGPx mutants showed that the introduction of Sec at the non−catalytic center noticeably decreased the GPx activity [32]. The high chemically reactive properties of Sec made the structure unstable. However, the formation of a diselenide bond between Sec132 and Sec8 overcame this negative effect and fixed the oligomerization loop which, in turn, contributed to the dimer and, consequently, the tetramer formation. Due to the maximum physiological concentration of GSH being no higher than 10 μM, the reduction ability of Sec−hGPx3−C8/132U was detected with a low concentration of GSH, using *t*−BuOOH as the oxidation substrate. As shown in Figure 6B, the Sec−hGPx3−C8/132U was able to reduce *t*−BuOOH at 10 μM GSH without the provision of another electron donor. Within four minutes, the oxidized NADPH was 5 nmol, as calculated from the decrease in the 340 nm absorbance from 0.0 to approximately −0.06.

### 3.5. Characterization of Sec−hGPx3−C8/132U

**Figure 7:**
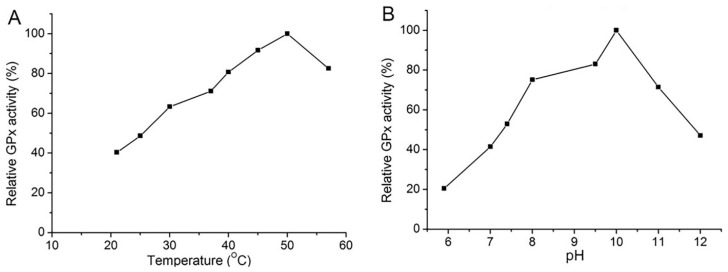
Effects of (**A**) temperature and (**B**) PH on the activity of Sec−hGPx3−C8/132U: Activity was determined with the concentrations of GSH and *t*−BuOOH at 1 mM and 0.3 mM, respectively. (**A**) Plot of GPx activity versus temperature; the highest activity at 50 °C was set as 100%; (**B**) Plot of GPx activity versus pH; the highest activity at pH 10 was set as 100%.

As shown in Figure 7, the activities of Sec−hGPx3−C8/132U were detected at temperatures between 20 °C and 56 °C, with the maximum activity at 50 °C, and 80% of the maximum activity was retained when the temperature was increased to 56 °C. This could also be explained by the properties of hGPx3 keeping the GPx activity stable when the structural conformation changed.

Its optimal pH was determined to be 10, and the relative activity was stable over a pH range of 8–11, even 41% of the maximum activity was retained at pH 12. The strongly increment in alkaline condition might due to the nucleophilic attack of selenium to one oxygen of the peroxide bond. Recent studies have elucidated the mechanism for the incredible efficiency of GPx [33,34,35]. The Sec reduces hydroperoxides through a dual attack on the peroxide bond in a two−step mechanism. First, a proton dislocation from the selenol to a close residue of the enzymatic pocket occurs, then, a nucleophilic attack of the anionic selenocysteine to one O atom takes place, while the proton shuttled back to the second O atom, promoting the formation of a water molecule [36,37,38,39].

**Figure 8:**
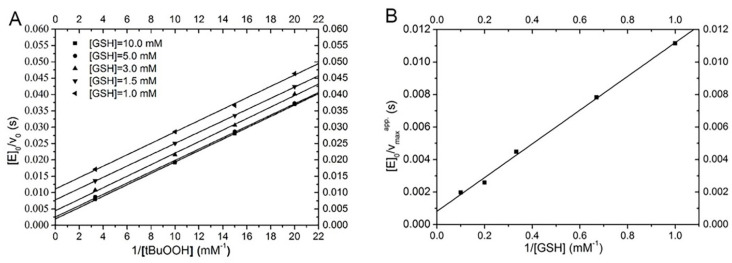
Double−reciprocal plots for the reduction of *t*−BuOOH with GSH catalyzed by Sec−hGPx3−C8/132U: (**A**) Single progression curves at 1.0 mM, 1.5 mM, 3.0 mM, 5.0 mM, and 10.0 mM GSH. (**B**) The apparent Vmax extrapolated for an infinite concentration of *t*−BuOOH was plotted against the reciprocal GSH concentrations.

Kinetic data for Sec−hGPx3−C8/132U were obtained by varying concentrations of *t*−BuOOH at several fixed GSH concentrations. Using double−reciprocal plots of [E_0_]/V versus the reciprocal concentrations of *t*−BuOOH at various fixed concentrations of GSH produced a set of parallel lines (Figure 8A); we found that Se−hGPx3−C8/132U follows a “ping pong” mechanism, as described by a Dalziel equation [40].

$$\;\frac{{\left[E\right]}_{0}}{V_{0}}=\frac{1}{k_{1}^{\prime }\left[\mathrm{tBuOOH}\right]}+\frac{1}{k_{2}^{\prime }\left[\mathrm{GSH}\right]}$$

In the secondary plot (Figure 8B), the apparent maximum velocities for infinite *t*−BuOOH concentrations were replotted against the reciprocal concentrations of GSH. The intercept of the secondary plot is not zero and represents the reciprocal value of the maximal velocity (k_cat_ = 964.3 ± 129.4 s^−1^). Thus, the Sec−hGPx3−C8/132U shows saturation kinetics. The rate constants *k*_1_ and *k*_2_ for *t*−BuOOH and GSH were (5.7 ± 0.30) × 10^5^ M^−1^ s^−1^and (8.1 ± 0.14) × 10^4^ M^−1^ s^−1^, respectively. The apparent K_m_ values for *t*−BuOOH and GSH were 1.69 mM and 11.9 mM, respectively, as calculated from K_m_ = k_cat_/*k*. This kinetic behavior is different from that of the classic hGPx1, which was reported to follow an unsaturated ping pong mechanism with infinite K_m_ and k_cat_ with GSH. The reason for this discrepancy is likely because of the missing Arg involved in GSH binding. In hGPx1, the guanidino groups of the four Arg residues surrounding the selenium contribute to the binding of GSH via electrostatic attraction, forcing the GSH molecule into an orientation that is appropriate for instant attack of the selenenic acid [15].

## 4. Discussion

We expressed an hGPx3 mutant, with all of the Cys residues changed to Ser, using a Cys auxotrophic strain of *E. coli* BL21(DE3)cys^−^ in our previous study [19]. Although lacking post−translational modification, the hGPx3 mutant still retained the ability to reduce H_2_O_2_, using GSH as an electron donor. In this study, the activity was not improved when the Cys residues were recovered, indicating that the three Cys residues in GPx3 were not important for the GPx activity, at least when using GSH as a redox donor. The previously reported GPx activity of hGPx3 purified from plasma was 20–25 U/mg [3,4,5], 75–110 U/mg [41], and 373 U/mg [26]. The activities of the mutants Sec−shGPx3−C77/132S and Sec−hGPx3−C8/132U were improved but still lower than that of hGPx3 purified from plasma, as reported previously. This may be due to the complicated intra−molecular disulfide bond formation and unglycosylated state of the recombinant hGPx3 and its mutants expressed in the *E. coli* expression system.

The failure to form a tetramer was also observed for Sec−hGPx3 in the condition of Cys recovery. The predicted disulfide bond between Cys8 and Cys132 is expected to fix the oligomerization loop between α3 and β4, which contributes to the formation of a tetramer [11,42]. The mutation analysis of Cys to Ser showed that the formation of intra−molecular disulfide bonds was very complicated. Cys132 in the highly flexible oligomerization loop tends to form a disulfide bond with Cys77, which was facilitated while Cys8 was mutated or deleted. The failure to correctly form a disulfide bond between Cys8 and Cys132 might increase the conformational freedom of the oligomerization loop.

The tendency to form disulfide or sulfhydryl–selenyl bonds in Sec−hGPx3 is ambiguous if the bridges have any physiological function. The formation of a disulfide bond between Cys77 and Cys132 is easily observed, especially when Cys8 is mutated or deleted, and the mutants retain GPx activity after conformational changes to form the disulfide bond. The formation might also occur under physiological conditions when the disulfide bond between Cys8 and Cys132 is reduced by reductants, such as Trx and GSH. The hGPx3 could efficiently use Trx as an electron donor while the concentration of GSH in the plasma was low and could not provide for the reduction of oxidized GPx3 [10]. The binding of GPx3 to the basement membranes increases the glutathione peroxidase activity at the binding site, and the locally secreted GSH serves as the reducing substrate [8,9]. Flexible, structural and conformational changes of GPx3 might be important for the binding process.

It is well−known that the Trx serve as the electron donor for CysGPx, forming intra− or inter−molecular disulfide bonds in the process of oxidation. The sequence similarity between hGPx3 and the non−seleno−containing hGPx5 leads us to believe that hGPx3 is evolutionarily related to CysGPx. The catalytic mechanism of CysGPx from plants was well−studied. The resolving Cys formed a disulfide bond with the oxidized active−site Cys [15,43], and Trx regenerated it to a reducing form. However, the formation of disulfide or sulfhydryl–selenyl bonds in recombinant Sec−hGPx3 and its mutants has no relationship with the in vitro catalytic process. The mechanism of hGPx3 using Trx as a redox donor, which might involve conformational flexibility, requires further study.

## 5. Conclusions

In conclusion, we utilized amber−less *E. coli* and tRNA^UTuT6^ to express Sec−hGPx3 with high yields. The recombinant Sec−hGPx3 formed complicated disulfide bonds, but failed to form a tetramer. SDS−PAGE analysis of Sec−hGPx3 variants indicated that the oligomerization loop was highly flexible and could transfer to form a disulfide bond between Cys77 and Cys132. The Sec−hGPx3−C8/132U exhibited high GPx activity after tetramer formation caused by diselenide bond formation.

## Figures and Tables

**Table 1:** Plasmids and *E. coli* strains used in this study.

Strain/Plasmid	Description	Source
Strains		
DH5α	Conventional host for plasmid propagation	Invitrogen
C321.ΔA.exp	Recoded E.coli MG1655 strain with all of its UAG codon depleted and UAG termination function removed	Marc Lajoie et al. [23] Addgene
Plasmids		
pN565	Expression of T7 RNA polymerase mutant	Christopher Voigt et al. [24] Addgene
pACYC−*selA/pstk*	Expression of SelA and PSTK	Dieter Söll et al. [20]
pGFIB−tRNA^UTuT6^	Expression of tRNA^UTuT6^	Jingyan Wei et al. [21]
pUC57−*hGPx3*	pUC57 with *hGPx3_49TGA_* fragment in BamHI/HindIII	This study
pRSF−*hGPx3_49TAG_* and its mutants	Expression of Sec−hGPx3 and its cysteine mutants	This study
pRSF−*hGPx3_49TCG_* and its mutants	Expression of hGPx3 (U49S) and its cysteine mutants	This study
pRSF−*shGPx_49TAG_* and its mutants	Expression of short isoform of Sec−shGPx3 and its cysteine mutants	This study
pRSF−*shGPx_49TCG_* and its mutants	Expression of short isoform of shGPx3 (U49S) and its cysteine mutants	This study

**Table 2:** Description of various mutant proteins in this study.

Protein	Description
Sec−hGPx3	Human GPx3 protein containing Sec49
Sec−hGPx3−C8S	Human GPx3 protein containing Sec49, Ser8
Sec−hGPx3−C8/77S	Human GPx3 protein containing Sec49, Ser8, Ser77
Sec−hGPx3−C8/132S	Human GPx3 protein containing Sec49, Ser8, Ser132
Sec−hGPx3−C77S	Human GPx3 protein containing Sec49, Ser77
Sec−hGPx3−C132S	Human GPx3 protein containing Sec49, Ser132
Sec−hGPx3−C77/132S	Human GPx3 protein containing Sec49, Ser77, Ser132
Sec−hGPx3C8/77/132S	Human GPx3 protein containing Sec49 Ser8, Ser77, Ser132
Sec−shGPx3	Short isoform of human GPx3 protein containing Sec49
Sec−shGPx3−C77S	Short isoform of human GPx3 protein containing Sec49, Ser77
Sec−shGPx3−C132S	Short isoform of human GPx3 protein containing Sec49, Ser132
Sec−shGPx3−C77/132S	Short isoform of human GPx3 protein containing Sec49, Ser77, Ser132
hGPx3 (U49S)/Ser−hGPx3	Human GPx3 protein containing Ser49
hGPx3 (U49S)−C8S/Ser−hGPx3−C8S	Human GPx3 protein containing Ser49, Ser8
hGPx3 (U49S)−C8/77S	Human GPx3 protein containing Ser49, Ser8, Ser77
hGPx3 (U49S)−C8/132S	Human GPx3 protein containing Ser49, Ser8, Ser132
hGPx3 (U49S)−C77S	Human GPx3 protein containing Ser49, Ser77
hGPx3 (U49S)−C132S	Human GPx3 protein containing Ser49, Ser132
hGPx3 (U49S)−C77/132S	Human GPx3 protein containing Ser49, Ser77, Ser132
hGPx3 (U49S)−C8/77/132S	Human GPx3 protein containing Ser49, Ser8, Ser77, Ser132
shGPx3 (U49S)	Short isoform of human GPx3 protein containing Ser49
shGPx3 (U49S)−C77S	Short isoform of human GPx3 protein containing Ser49, Ser77
shGPx3 (U49S)−C132S	Short isoform of human GPx3 protein containing Ser49, Ser132
Sec−hGPx3−C8/132U	Human GPx3 protein containing Sec49, Sec8, Sec132

## Data Availability

The data are contained within the article and supplementary materials.

## Supplementary Materials

The following supporting information can be downloaded at: https://www.mdpi.com/article/10.3390/antiox11061083/s1. Table S1. Primer sequences used for cloning and mutation; Figure S1. SDS−PAGE analysis of shGPx3 (U49S) and shGPx3 (U49S) mutants under non−reducing conditions.
