# Supplementary material for: Reactive Human Plasma Glutathione Peroxidase Mutant with Diselenide Bond Succeeds in Tetramer Formation
Source: Antioxidants (Basel). 2022 May 29;11(6):1083. doi: 10.3390/antiox11061083 (PMC9220127; doi:10.3390/antiox11061083)
Supplement: Supplementary file 1 [file antioxidants-11-01083-s001.zip › antioxidants-1713817-supplementary.pdf]

Reactive human plasma glutathione peroxidase mutant with Diselenide Bond Succeeds in tetramer formation

Zhenlin Fan[1,†], Qi Yan[1,†], Jian Song[2,*] and Jingyan Wei[1,3,*]

[1] College of Pharmaceutical Science, Jilin University, Changchun 130021, China; fanzl11@mails.jlu.edu.cn (Z.F.); qiyan20@mails.jlu.edu.cn (Q.Y.)

[2] College of Electronic Science and Engineering, Jilin University, Changchun 130000, China

[3] Key Laboratory for Molecular Enzymology and Engineering of the Ministry of Education, Jilin University, Changchun 130000, China

* Correspondence: songjian13@mails.jlu.edu.cn (J.S.); weijy@jlu.edu.cn (J.W.); Tel.: +86-431-8561-9716 (J.W.); Fax: +86-431-8561-9252 (J.W.)

† These authors contributed equally to this work.

Table S1. Primer sequences used for cloning and mutation.

Primers	Sequence (5'−3')
TAG−F	CAACGTGGCCAGCTACTAGGGCCTGACGGGCCAGTAC
TAG−R	GTACTGGCCCGTCAGGCCCTAGTAGCTGGCCACGTTG
TCG−F	CAACGTGGCCAGCTACTCGGGCCTGACGGGCCAGTAC
TCG−R	GTACTGGCCCGTCAGGCCCGAGTAGCTGGCCACGTTG
C8S−F	GAAGTCGAAGATGGACTCGCATGGTGGCATAAGTGG
C8S−R	CCACTTATGCCACCATGCGAGTCCATCTTCGACTTC
C77S−F	CATTCTGGGCTTTCCCTCGAACCAATTTGGAAAACAGG
C77S−R	CCTGTTTTCCAAATTGGTTCGAGGGAAAGCCCAGAATG
C132S−F	CTTTCCTAAAGAACTCCTCGCCTCCCACCTCGGAGCTCC
C132S−R	GGAGCTCCGAGGTGGGAGGCGAGGAGTTCTTTAGGAAAG
shGPx3−F	CGGGATCCGGGCACCATTTACGAG
hGPx3−R	CCCAAGCTTTTACTTCCTCTTGACC
C8U−F	GAAGTCGAAGATGGACTAGCATGGTGGCATAAGTGG
C8U−R	CCACTTATGCCACCATGCTAGTCCATCTTCGACTTC
C132U−F	CTTTCCTAAAGAACTCCTAGCCTCCCACCTCGGAGCTCC
C132U−R	GGAGCTCCGAGGTGGGAGGCTAGGAGTTCTTTAGGAAAG

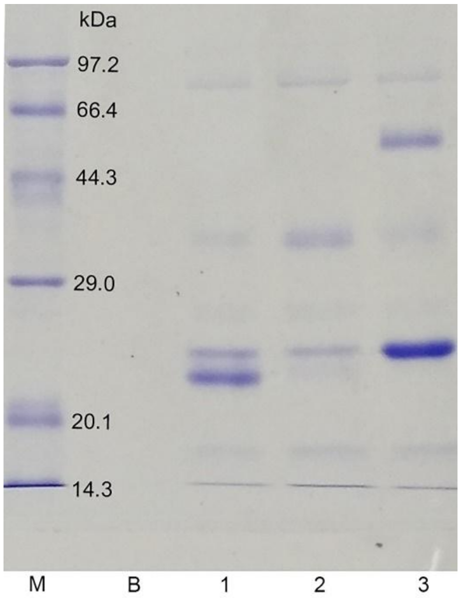

Figure S1: SDS−PAGE analysis of shGPx3 (U49S) and shGPx3 (U49S) mutants under non−reducing conditions without boiling treatment. Lanes are as follows: M, marker; B, blank; 1, non−reduced shGPx3 (U49S); 2, non−reduced shGPx3 (U49S)−C77S; 3, non−reduced shGPx3 (U49S)−C132S.
